# Leiomyosarcoma of the sigmoid colon with multiple liver metastases and gastric cancer: a case report

**DOI:** 10.1186/1471-230X-12-98

**Published:** 2012-07-31

**Authors:** Yoichi Hamai, Jun Hihara, Manabu Emi, Yoshiro Aoki, Kei Kushitani, Kazuaki Tanabe, Morihito Okada

**Affiliations:** 1 Department of Surgical Oncology, Research Institute for Radiation Biology and Medicine, Hiroshima University, 1-2-3 Kasumi, Minami-ku, Hiroshima 734-8551, Japan; 2 Department of Pathology, Graduate School of Biomedical Sciences, Hiroshima University, Hiroshima Japan; 3 Department of Surgery, Division of Frontier Medical Science, Graduate School of Biomedical Sciences, Hiroshima University, Hiroshima Japan

**Keywords:** Leiomyosarcoma, Gastric cancer, Liver metastasis, Surgery, Chemotherapy

## Abstract

**Background:**

Leiomyosarcoma (LMS) of the gastrointestinal tract is an extremely rare high-grade neoplasm with poor prognosis. For advanced LMS with distant metastasis, the decision as to the choice of the most appropriate therapeutic strategy, including chemotherapy and surgery, is difficult. Here, we present an unusual case of LMS of the sigmoid colon with liver metastases and gastric cancer. The survival of this patient was prolonged by a combined modality therapy involving chemotherapy and surgery.

**Case presentation:**

A 66-year-old woman who had been diagnosed with advanced gastric cancer and multiple liver metastases was referred to our hospital. The initial treatment with docetaxel and S-1 considerably reduced both the gastric cancer and liver tumors; consequently we performed surgical resection. Pathological examination revealed that no viable tumor cells remained in the stomach and chemotherapy resulted in complete remission of the gastric cancer. The liver tumors were immunohistochemically diagnosed as LMS. A tumor of the sigmoid colon was subsequently discovered and the liver tumors were found to have recurred. The surgically resected sigmoid colon and liver tumors were all immunohistochemically diagnosed as LMS. These findings indicated that the multiple liver metastases arose from the LMS in the sigmoid colon, and that they were accompanied by advanced gastric cancer. We performed another surgical resection and administered chemotherapy to treat the recurring liver metastases. The patient survived for 4 years and 10 months after initial presentation at our hospital.

**Conclusion:**

Colonic LMS is rare and its joint occurrence with gastric cancer is extremely unusual. Although LMS is a high-grade neoplasm, a multimodal therapeutic approach can increase patient survival time even when multiple liver metastases are present.

## Background

Leiomyosarcoma (LMS) of the gastrointestinal (GI) tract is extremely rare, and only a few reports have been published in reviews of GI mesenchymal tumors [1,2]. LMS frequently metastasizes to the liver and has a poor prognosis. Unlike gastrointestinal stromal tumors (GIST) effective molecular therapy is not available for LMS. Thus, the decision regarding the selection of an optimal therapeutic strategy for advanced LMS with metastasis is difficult [1,3]. In the present case report, we describe a 66-year-old woman with LMS of the sigmoid colon accompanied by multiple liver metastases and advanced gastric cancer. The survival of this patient was prolonged by a combination of three surgical resections and chemotherapy.

## Case presentation

**Figure 1:**
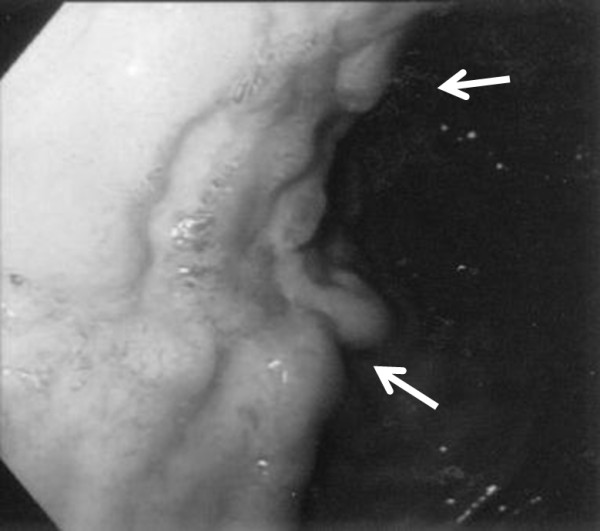
**Gastrointestinal fiberscope findings upon admission of the patient. **The arrows indicate the position of ulcerative lesions on the anterior wall of the gastric corpus.

**Figure 2:**
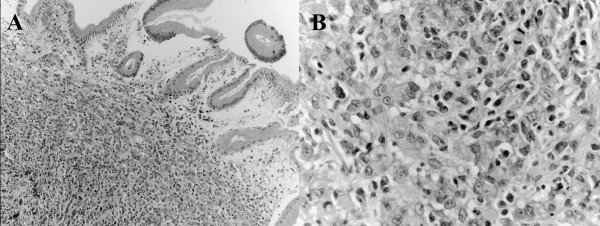
**Microscopic findings from a biopsy specimen resected from an ulcerative lesion. **Poorly differentiated adenocarcinoma is evident. H&E stain × 100 (**A**) and × 200 (**B**).

**Figure 3:**
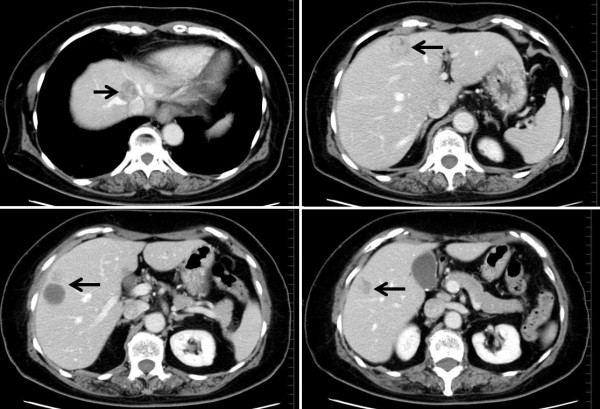
**Abdominal contrast-enhanced CT findings upon admission. **Arrows indicate the positions of four heterogeneous liver tumors.

A 66-year-old woman diagnosed with advanced gastric cancer and multiple liver metastases was referred to our hospital in March 2003. She was not a carrier of the hepatitis virus, or an alcoholic with previous hepatic disease. Furthermore, she did not have a family history of malignant neoplasia. Gastrointestinal fiberscopy upon admission showed an irregular ulcerative lesion on the anterior wall of the gastric corpus (Figure 1), and the pathological diagnosis from the biopsy specimens was poorly differentiated adenocarcinoma (Figure 2A, B). Abdominal computed tomography (CT) images revealed four space-occupying lesions with diameters of 2–3 cm with internal heterogeneity due to a relative lack of effect of the contrast medium in the liver (Figure 3). This suggested the presence of metastasis from the gastric cancer and no enlarged lymph nodes around the stomach. A barium enema revealed no evidence of dissemination or colon tumors. Based on these findings, the patient was diagnosed with Stage IV gastric cancer with hematogenous metastases according to the Japanese Classification of Gastric Carcinoma [4].

We administered chemotherapy with docetaxel and oral S-1 to treat the gastric cancer and liver metastases [5]. Seventeen cycles of this regimen over a period of one year considerably reduced the gastric cancer and liver tumors, and new cancerous lesions did not appear. However, adverse effects prevented the patient from continuing with this regimen. We considered that all of the lesions were completely resectable at this point, and the patient provided written informed consent to proceed with surgery at 14 months after starting chemotherapy.

**Figure 4:**
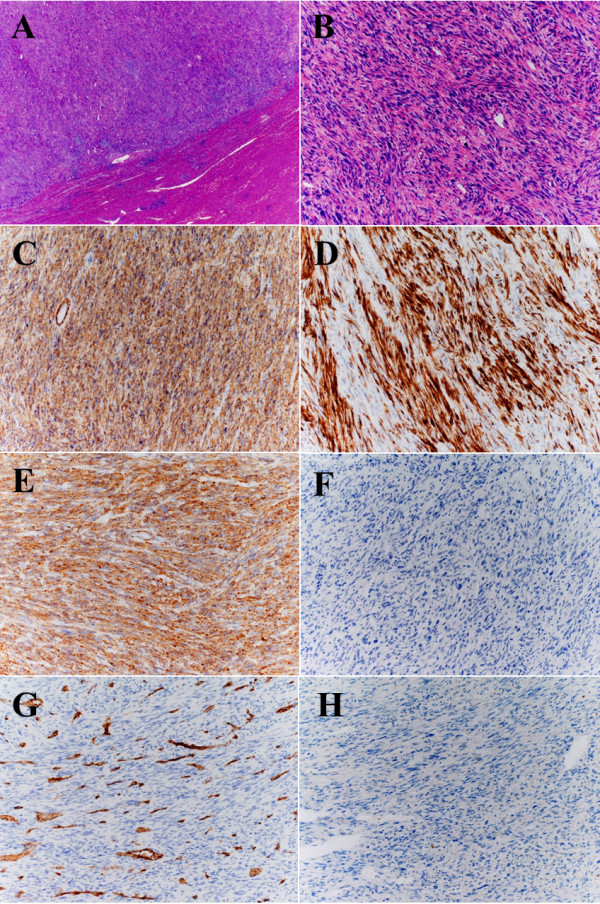
**Microscopic findings from a resected liver specimen. **Tumor proliferation is evident adjacent to scar tissue (**A**) and has intersecting fascicles of atypical spindle-cells (**B**). The tumor was immunohistochemically positive for SMA (**C**), desmin (**D**) and h-caldesmon (**E**), and negative for c-KIT (**F**), CD34 (**G**) and S-100 (**H**). The blood vessel wall was positive for CD34 (**G**). A and B, H&E; C, SMA; D, desmin; E, h-caldesmon; F, C-kit; G, CD34; and H, S-100.

Ascites and peritoneal disseminated lesions were not evident during the procedure, which included total gastrectomy and partial liver resections for all liver tumors. Pathological examination revealed that no viable tumor cells remained in the stomach and chemotherapy resulted in complete remission of the gastric cancer. The liver tumors were immunohistochemically positive for smooth muscle actin (SMA), desmin and h-caldesmon, and negative for c-KIT, CD34 and S-100 (Figure 4). Furthermore, an average of 20 mitoses per 10 high power fields was observed in the liver tumors, which were diagnosed as being LMS with high mitotic activity. Until that point, we considered that the liver tumors were metastases that had developed from the gastric cancer. However, the histological type of the liver tumors was LMS and not adenocarcinoma. Thus, we had to assume that the liver LMSs were primary tumors that developed from liver or metastatic tumors from an unknown primary LMS.

**Figure 5:**
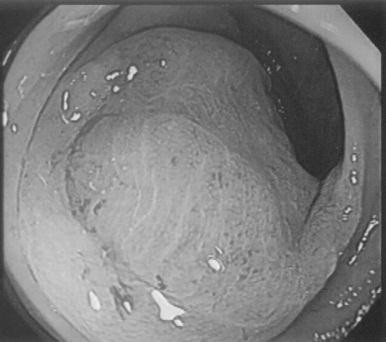
**Colonoscopy findings. **The lesion can be seen protruding from the submucosa.

We administered only oral S-1 as adjuvant chemotherapy after the surgery. However, 11 months after this treatment, four liver tumors developed and we changed the chemotherapy to irinotecan and cisplatin. This regimen was ineffective and the liver tumors gradually enlarged. Furthermore, a tumor-like mass in the sigmoid colon was incidentally discovered on CT scan and positron emission tomography/computed tomography (PET-CT) during this therapeutic process; this gradually increased in size to 3 cm in diameter. Colonoscopy revealed a lesion protruding from the submucosa with a normal mucosal surface (Figure 5). Although we suspected that this tumor might be a solitary peritoneal metastasis from gastric cancer, our assessment was that complete resection of the colon tumor and all of the liver tumors would be feasible.

**Figure 6:**
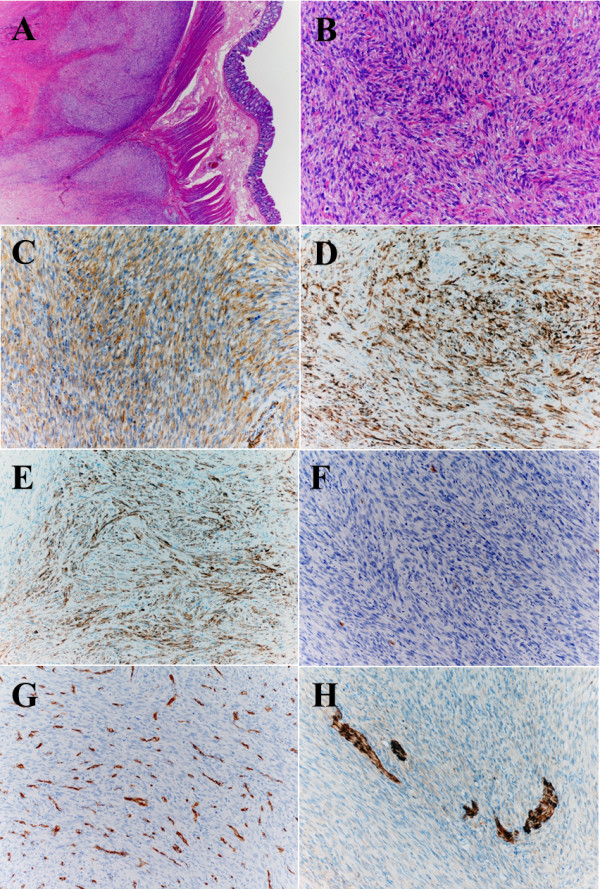
**Microscopic findings from a resected colon specimen. **The tumor developed from the muscular layer of the colon (**A**). Histological and immunohistochemical findings for the colon and liver tumors were similar (**B-H**). The blood vessel wall and nerve tissues were positive for CD34 (G) and S-100 (H), respectively. **A** and **B**, H&E; **C**, SMA; **D**, desmin; **E**, h-caldesmon; **F**, C-kit; **G**, CD34; and **H**, S-100.

A second surgical resection of the four liver tumors and a partial colon resection proceeded at 2 years and 5 months after the commencement of first-line chemotherapy. The sigmoid colon and liver tumors were all immunohistochemically diagnosed as LMS (Figure 6). This finding indicated that the LMS had originated in the sigmoid colon, and that the multiple liver metastases had arisen from a colonic LMS that was accompanied by advanced gastric cancer at the time of the first admission.

The patient underwent a third liver resection to treat two new liver metastases that developed at 7 months after the second procedure. This was because we considered that there might be only a limited chance of a good response to chemotherapy in the case of the LMS, and all of the liver metastases were also completely resectable. Thereafter, despite ifosfamide and epirubicin chemotherapy followed by dacarbazine, multiple liver and lung metastases eventually developed and the patient died at 4 years and 10 months after the first presentation at our hospital.

## Conclusions

The incidence of LMS in the GI tract is extremely rare and GIST account for most GI mesenchymal tumors. Recent reports describe the classification of only three among 262 GI mesenchymal tumors as being LMS [1], and only seven out of 253 mesenchymal tumors as being involved with the colon [2]. Furthermore, the reported incidence of LMS is 3–6% among GI mesenchymal tumors in other regions of the GI tract such as the esophagus, duodenum and anorectum [6-8].

The reported clinical features of LMS of the GI tract are gross polypoid and intramural types that can arise from either the muscularis mucosae or the propria [1,2,9]. Neighboring tissue infiltration and liver metastases are common, but lymphogenic spread is rare [1,9]. LMSs are extremely high-grade neoplasms with high mitotic activity, and patient survival time is usually short [2,6-8]. The barium enema and CT scan did not detect LMS of the sigmoid colon in our patient at the first admission. This was because the intramural tumor was probably very small and hidden in the colonic wall. However, the LMS had high mitotic activity and had already caused multiple liver metastases by that time. In addition, the liver metastasis had subsequently recurred despite complete resection of the primary site and the initial liver metastases. LMS seems to have a very high hematogenous metastatic potential.

**Figure 7:**
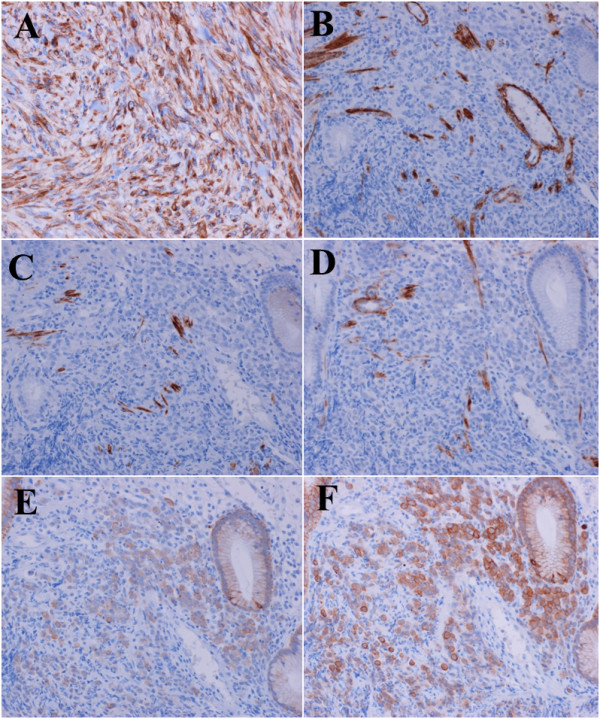
**Additional immunohistochemical analyses of liver and gastric tumor. **The liver tumor was immunohistochemically positive for vimentin (**A**). The gastric tumor was negative for SMA (**B**), desmin (**C**) and h-caldesmon (**D**), and positive for AE1/AE3 (**E**) and CAM5.2 (**F**). The blood vessel wall and fibrous tissue were positive for SMA (B), desmin (C) and h-caldesmon (D). **A**, vimentin; **B**, SMA; **C**, desmin; **D**, h-caldesmon; **E**, AE1/AE3; and **F**, CAM5.2.

The histogenetic, clinicopathological and immunohistochemical profiles of LMS and GIST differ [1]. Whereas GIST arises from the interstitial cell of Cajal, LMS originates from smooth muscle cells within the muscularis mucosa or muscularis propria [1,9], and it lacks *KIT* mutations [2,6,7]. An immunohistochemical analysis is essential for a definitive diagnosis of LMS, which is regularly negative for c-kit and CD34 and positive for smooth muscle markers such as actin, desmin and h-caldesmon [10,11]. The immunohistochemical findings of specimens obtained from our patient after resection of the colon and liver tumors were similar. Furthermore, the specimens were also positive for vimentin in additional immunohistochemical analysis (Figure 7A). This combination of highly-specific immunohistochemical findings provided a definitive diagnosis of colon LMS and multiple liver metastases.

Moreover, in the diagnosis of the present case, LMSs in both the liver and colon were possibly metastatic tumors that originated from primary LMS in another organ. Actually, LMS of the left thigh that metastasized to the liver, large bowel and lymph nodes has been reported [12]. However, in the present study, soft tissue tumor was not detected using periodically-performed CT and PET-CT scans in the course of treatment. Furthermore, we recently performed additional immunohistochemical analyses for gastric lesions to distinguish them from LMS. The biopsy tissue of the gastric lesion was immunohistochemically negative for desmin, SMA and h-caldesmon, and positive for cytokeratin (AE1/AE3 and CAM5.2) (Figure 7B-F). The LMS and gastric lesions represented a completely different histological picture and immunohistochemical profiles. Thus, we could conclude that this case represents a combination of colonic LMS that metastasized to the liver and gastric cancer.

Surgical resection is the most frequent approach to treating LMS [2,6-8]. One report describing metastatic sarcoma to the liver, that also included liver metastases from GIST and extra-intestinal LMS, has shown that the complete resection of liver metastases from the sarcoma was associated with prolonged survival, and that the interval to metachronous metastasis was an independent predictor of outcome [13].

Chemotherapy generally plays a limited role in the treatment of LMS [13,14]. Furthermore, a specific molecular therapy is currently available for GIST, but not for LMS. Reports indicate that 30–60% clinical response rates can be achieved in the treatment of LMS using combinations of docetaxel and gemcitabine [15], and in the treatment of advanced soft tissue sarcoma using ifosfamide with anthracycline and/or dacarbazine [16-18]. Only first line chemotherapy with docetaxel and S-1 was found to be effective against both the gastric cancer and LMS in our patient. The overall response rate of this regimen is reported to be 56.3% for gastric cancer [5]. It was considered that docetaxel, which is so frequently used and effective against both LMS and gastric cancer, could simultaneously reduce the size of these tumors.

We initially considered that the liver tumors were metastases arising from gastric cancer. However, we resected the stomach and liver tumors because all of the tumors remained small. They proved to be completely resectable and de novo lesions did not appear during the first year of chemotherapy. Had the first chemotherapy regimen been ineffective and had the number of liver tumors and/or the size of the gastric cancer increased, surgical resection in our patient would not have been indicated. This was because of the fact that these factors would have pointed to a decreased probability of complete resection being achievable.

The tumorigenesis of gastric cancer and LMS has been reported to involve various factors [19-22]. Common factors, such as infection with the Epstein-Barr virus and molecular alterations in *RASSF1A*, were also indicated in the occurrence of these tumors [23,24]. Furthermore, in an experimental model, simultaneous exposure to both nitrosoguanidine and acetylsalicylic acid caused synchronous development of both gastric cancer and LMS [25]. Intragastric application of N-methylnitrosourea also revealed increased susceptibility to chemical tumorigenesis of gastric cancer and sarcoma in p53 knockout mice [26]. In the present case, it is not clear if the association is a simple coincidental coexistence or if the two types of lesion are connected by a causal relationship that might involve a common aetiology and tumorigenic mechanisms.

Colonic LMS is rare and its occurrence in combination with gastric cancer is extremely unusual. Although the diagnosis in our patient was complicated by the presence of both gastric cancer and LMS, an immunohistochemical study of surgical specimens confirmed the final diagnosis of LMS of the sigmoid colon with multiple liver metastases. We concluded that the multimodal approach comprising chemotherapy and complete surgical resection controlled the LMS, even with multiple liver metastases present, and improved the survival of this patient.

## Consent

Written informed consent was obtained from one of the patients’ relatives for publication of this case report and the accompanying images. A copy of the written consent is available for review by the Series Editor of this journal.

## Abbreviations

CT: Computed tomography; GI: Gastrointestinal; GIST: Gastrointestinal stromal tumor; LMS: Leiomyosarcoma; PET-CT: Positron emission tomography/computed tomography; SMA: Smooth muscle actin.

## Competing interests

The authors declare that they have no competing interests.

## Authors’ contributions

All of the authors have read and approved the final manuscript. Dr YH was responsible for the design and drafting of the manuscript; Drs JH and KT were responsible for the conception and revision of the manuscript, and also the pathological diagnosis; Drs ME and YA carried out the surgical operation and clinical management of the patient; Dr KK undertook the pathological diagnosis; and Dr MO was responsible for the final review and revision of the manuscript and the supervision of the study.

## Pre-publication history

The pre-publication history for this paper can be accessed here:

http://www.biomedcentral.com/1471-230X/12/98/prepub
